# The complete mitochondrial genome of Eaton’s skate, *Bathyraja eatonii* (Rajiformes, Arhynchobatidae)

**DOI:** 10.1080/23802359.2020.1847608

**Published:** 2021-01-12

**Authors:** Jinmu Kim, Sung-Min Jang, Eunkyung Choi, Euna Jo, Seung Jae Lee, Sun Hee Kim, Young Min Chi, Jin-Hyoung Kim, Hyun Park

**Affiliations:** a Division of Biotechnology, College of Life Sciences and Biotechnology, Korea University, Seoul, Korea;; b Unit of Research for Practical Application, Korea Polar Research Institute (KOPRI), Incheon, Korea;; c Greenwitch Co, Chungcheongbuk-do, Korea

**Keywords:** PacBio, mitochondria genome, *Bathyraja eatonii*, long-read technology, Arhynchobatidae

## Abstract

The complete mitochondrial genome of Eaton’s skate *Bathyraja eatonii* was studied using the long-read technology, PacBio Sequel System. The complete mitochondrial genome form of *B. eatonii* was 16,698 bp and it’s comprised of 13 protein-coding genes, 22 tRNA and 2 rRNA. The base composition of *B. eatonii* is analyzed 31.94% for A, 33.94% for T, 13.49% for G, 20.64% for C, the result of GC content was 33.94%. Phylogenetic analysis showed that *B. eatonii* was closely related to *Bathyraja meridionalis* in Arhynchobatidae family, and this first mitochondrial genome of Antarctic skate would provide fundamental information to the evolutional relationship of Antarctic fishes

Cartilaginous fish are unique that have a skeleton made of cartilage rather than bone. For example, *Bathyraja eatonii* (Eaton’s skate) is one of them. Expect the shark species, in the Antarctic region, there are six species of *Bathyraja*. *Bathyraja eatonii* lives in the Southern Ocean, around the Antarctic Peninsula, and they are common on the Kerguelen Plateau around South Shetland islands and South Orkney (Verde et al. 2005). There are eight complete genomes of mitochondria in *Bathyraja* genus, but the complete mitogenome of *B. eatoni* is not published yet. So, the complete mitochondria genomes of *Bathyraja* genus were used as reference for classifying and making a phylogenetic tree.

The sample was collected from Southern ocean (65°05′S, 170°30′E on CCAMLR Subarea 88.1), Antarctica, and the specimen was deposited at the Earth Biocollection in the Division of Biotechnology, Korea University with accession number KAN0016030. DNA was isolated using the conventional phenolchloroform method. After the extraction, G-tube (Covaris, CA, USA) shearing device and BluePippin system (Sage Science, MA, USA) were used for making a 20 kb size-selected templates according to the recommended protocol. Using the PacBio Sequel platform according to the SMRTbell™ Template Prep Kit 1.0 (Pacific Biosciences, Menlo Park, CA, USA) manufacturer’s protocol, SMRTbell library was produced. The PacBio subreads for mitochondria genome were used to filtered out by using 16 s rRNA and CO1 gene sequences of other related Antarctic fishes. Then, CANU assembler (Koren et al. 2017) was used for *de novo* assembly and the assembled mitogenomic sequences were annotated by MITOs web server (Bernt et al. 2013).

The complete mitogenome of *B. eatonii* (GenBank Number: MT447072) was 16,698 bp in length and it includes 13 protein-coding genes, 22 transfer RNAs and two ribosomal RNAs. The base composition *B. eatonii* is estimated 31.94% for A, 33.94% for T, 13.49% for G, 20.64% for C, and the result of GC content was 33.94%. (https://jamiemcgowan.ie/bioinf/gc.html). The relationship between *B. eatonii* was analyzed with 11 species in Arhynchobatidae family, 4 species in Rajidae family, and 4 species in Nototheniidae family using 13 protein-coding genes (Figure 1). Maximum-likelihood (ML) tree was built with 1000 bootstrap replications and JTT matrix-based model (Jones et al. 1992) by MEGA X software (Kumar et al. 2018). Looking at phylogenetic tree, *B. eatonii* is closely related to *Bathyraja meridionalis* and they were included with other species in Arhynchobatidae family.

**Figure 1.**
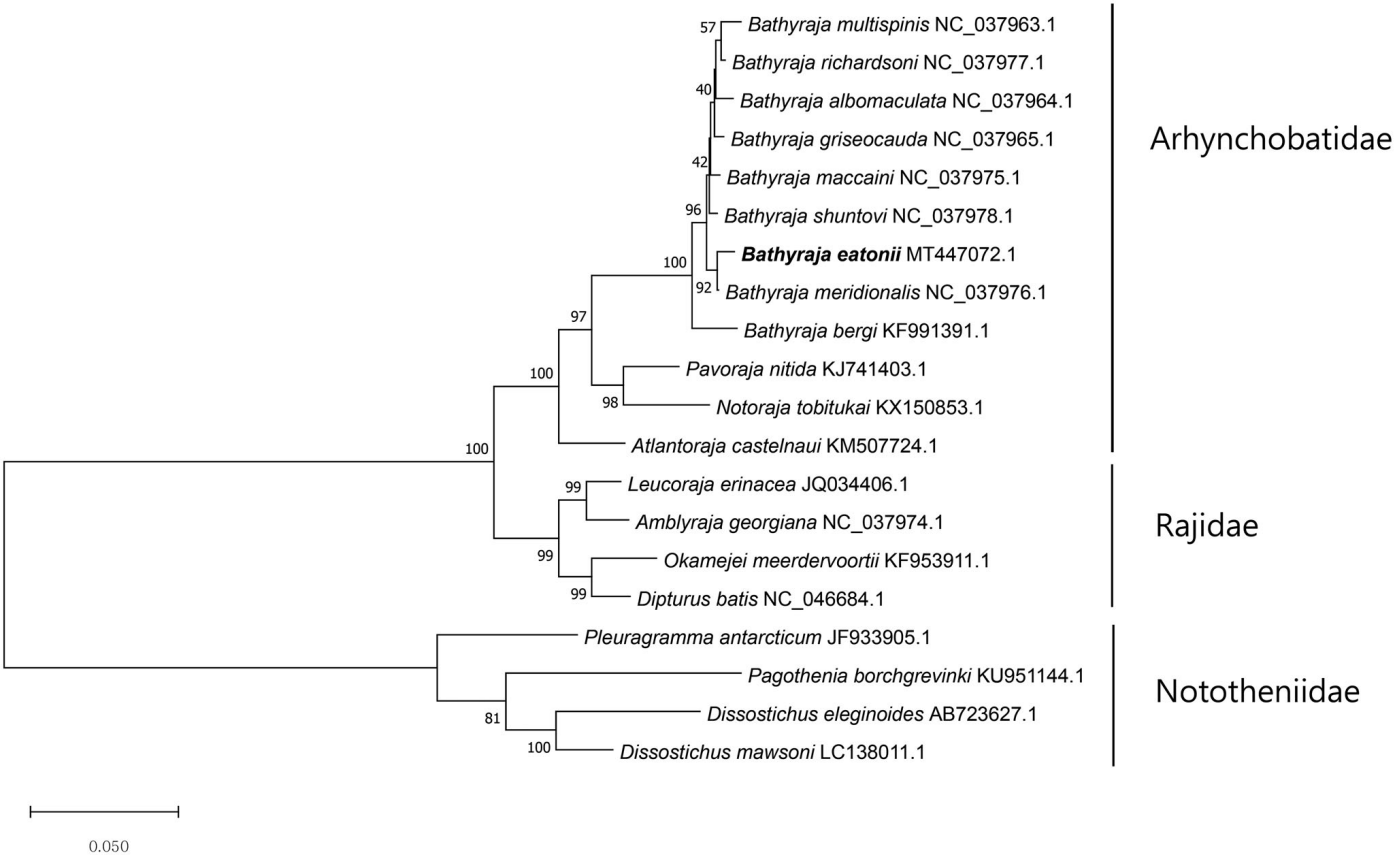
Using MEGA X software, maximum Likelihood method and JTT matrix-based, phylogenetic tree was built with 20 species in Arhynchobatidae, Rajidae, Nototheniidae families including *B. eatonii*. The GenBank number and scientific name were included for each species and *B. eatonii* is bolded.

## Data Availability

The data that support the findings of this study are openly available in NCBI under the accession MT447072 (https://www.ncbi.nlm.nih.gov/nuccore/MT447072.1/).

## References

[CIT0001] Bernt M, Donath A, Jühling F, Externbrink F, Florentz C, Fritzsch G, Pütz J, Middendorf M, Stadler PF. 2013. MITOS: improved de novo metazoan mitochondrial genome annotation. Mol Phylogenet Evol. 69(2):313–319. 22982435 10.1016/j.ympev.2012.08.023

[CIT0002] Jones DT, Taylor WR, Thornton JM. 1992. The rapid generation of mutation data matrices from protein sequences. Comput Appl Biosci. 8(3):275–282. 1633570 10.1093/bioinformatics/8.3.275

[CIT0003] Koren S, Walenz BP, Berlin K, Miller JR, Bergman NH, Phillippy AM. 2017. Canu: scalable and accurate long-read assembly via adaptive k-mer weighting and repeat separation. Genome Res. 27(5):722–736. 28298431 10.1101/gr.215087.116 PMC5411767

[CIT0004] Kumar S, Stecher G, Li M, Knyaz C, Tamura K. 2018. MEGA X: molecular evolutionary genetics analysis across computing platforms. Mol Biol Evol. 35(6):1547–1549. 29722887 10.1093/molbev/msy096 PMC5967553

[CIT0005] Verde C, De Rosa MC, Giordano D, Mosca D, De Pascale D, Raiola L, Cocca E, Carratore V, Giardina B, Di Prisco G, et al. 2005. Structure, function and molecular adaptations of haemoglobins of the polar cartilaginous fish *Bathyraja eatonii* and *Raja hyperborea*. Biochem J. 389(2):297–306. 15807670 10.1042/BJ20050305 PMC1175106

